# Editorial: The Treatment of RAS or BRAF Mutated Metastatic Colorectal Cancer: Challenges and Perspectives

**DOI:** 10.3389/fonc.2022.852445

**Published:** 2022-03-17

**Authors:** Alessandro Ottaiano, Massimiliano Berretta, Claudia Von Arx, Monica Capozzi, Michele Caraglia

**Affiliations:** ^1^Istituto Nazionale Tumori di Napoli, Istituto di Ricovero e Cura a Carattere Scientifico (IRCCS) “G. Pascale”, Naples, Italy; ^2^Department of Clinical and Experimental Medicine, University of Messina, Messina, Italy; ^3^Department of Precision Medicine, University of Campania “L. Vanvitelli”, Naples, Italy

**Keywords:** colorectal cancer, KRAS, BRAF, prognosis, therapy

Metastatic colorectal cancer (mCRC) is the third cause of cancer-related death worldwide and new therapeutic strategies are needed to ameliorate survival of patients. Improvements in basic research and gene assessment techniques are leading to unexpected therapeutic opportunities. The intent of this Research Topic was to prompt researchers to deep the insight into prognostic and therapeutic issues regarding mCRC patients bearing RAS or BRAF mutations. Seven articles were selected for publication. We are grateful to Authors and Editors who contributed to this difficult issue. The articles had specific informative and methodologic strengths; limits were well described and highlighted in discussions allowing correct interpretation of data. Rosati et al., Grassi et al., and Takeda and Sunakawa presented extensive and critical literature reviews showing the “state of the art” as well as the therapeutic perspectives of RAS/BRAF mutated mCRC patients. Their research highlighted how the treatment of RAS/BRAF mutated mCRC patients is changing from an aggressive chemotherapy approach (“triplets” with or without biologic agents) to combination of target-based (MEK, PIK3CA, WEE1 inhibitors, anti-EGFR drugs, *etc.*) and immunological drugs (spartalizumab, nivolumab, *etc.*). Therefore, chemotherapy has been limited to a marginal role. This is a therapeutic switch, which will involve most cancers in the next future. Furthermore, new data have recently ameliorated the pessimistic opinions about the difficult drugging of mutated-RAS. First, the FDA approval of sotorasib (small inhibitor of mutated KRAS) in KRAS p.G12C-mutated advanced NSCLC (Non-Small Cell Lung Cancer) previously treated with standard therapies based on the results of a large phase II study reporting long-lasting both complete and partial responses (response rate: 37.1%, median duration of response 11.1 months). Second, the data from KRISTAL-1 study showing that adagrasib (another p.G12C KRAS inhibitor) is well tolerated and active in combination with cetuximab (response rate: 43%) in heavily pre-treated p.G12C RAS mutated mCRC patients. Third, the FOCUS-4 study where adavosertib (small inhibitor of WEE1 kinase, a regulator of G2 to M cell cycle transition by phosphorylation of CDK1) significantly prolonged progression-free survival versus active monitoring of about 2 months (hazard ratio: 0.35; 95% confidence intervals: 0.18-0.68; *P*=0.0022) in mCRC patients bearing both RAS and TP53 mutations.

It is increasingly clear that RAS/BRAF mutated patients are not homogeneous populations on a prognostic and therapeutic point of views. In this context, Guan et al. report both prognostic and predictive significance of differential p.V600E BRAF and non-p.V600E mutations in 74 BRAF mutated patients. This is a relatively large population considering that previous studies were conducted in smaller subgroups (see also Table 1 of Takeda and Sunakawa providing readers with an excellent descriptive view of past, present and future). They show that patients with non-V600E mutations had longer survival than those with typical p.V600E variant. Therefore, the non-V600E mutated CRC patients may be a subtype of CRC with specific still unknown biologic characteristics. Moreover, they evidence that the dismal prognosis of p.V600E mutation could be related to loss of CDX2 (a homeobox transcription factor playing crucial role in the differentiation of the digestive system cells). With the last observation, these authors add originality and complexity to BRAF mutational assessment in our Research Topic. We have to greatly thank Padder et al. who present an interesting and original article about the connection from a BRAF-driven oncogenic signal with mitochondrial dynamics. The authors show that BRAF-mutated tumor cells are more prone to mitochondria fragmentation conferring glycolytic (metabolic reprogramming) and metastatic (EMT -Epithelial-to-Mesenchymal Transition- and growth advantage) phenotype. This phenomenon is in part dependent on DRP1/PDK1 (Dynamin Related Protein1/Pyruvate Dehydrogenase Kinase 1) activity. DRP1 inhibition could reduce mitochondrial fission and consequent neoplastic phenomena even if the occurrence of other effects, such as mitophagy, cannot be excluded. Finally, we really appreciated two large prognostic studies by Cao et al. and Zhao et al. The first one reported data from an impressive sample of 9,736 patients establishing and validating a nomogram based on clinico-pathological characteristics for predicting cancer specific and overall survival in patients with CRC liver metastases. The second one, reported data on the predictive value of KRAS and MMR (Mismatch Repair) status of combining serum tumor markers level with basal clinic-pathological characteristics in 2,279 patients. These prognostic tools were developed with a strong practical and social approach. In fact, the merit of the authors was to provide a “convenient, non-invasive and cost effective modality to identify appropriate candidates for genetic testing” “…in developing countries, especially in county-level hospitals”.

The development and availability of techniques based on deep sequencing and of repository data bases with the genetic information of the patients represent a new landscape in the definition of both new diagnostic/prognostic markers and therapeutic targets in the frame of the precision medicine.

On this view, the self-tailoring of the treatment for an individual patient is changing from a dream to a fact.

## Author Contributions

All the authors are Topic Editor: The Treatment of RAS or BRAF Mutated Metastatic Colorectal Cancer: Challenges and Perspectives. All authors contributed to the article and approved the submitted version.

## Conflict of Interest

The authors declare that the research was conducted in the absence of any commercial or financial relationships that could be construed as a potential conflict of interest.

## Publisher’s Note

All claims expressed in this article are solely those of the authors and do not necessarily represent those of their affiliated organizations, or those of the publisher, the editors and the reviewers. Any product that may be evaluated in this article, or claim that may be made by its manufacturer, is not guaranteed or endorsed by the publisher.

